# Proteogenomic analysis of melanoma brain metastases from distinct anatomical sites identifies pathways of metastatic progression

**DOI:** 10.1186/s40478-020-01029-x

**Published:** 2020-09-05

**Authors:** Erin M. Taylor, Stephanie D. Byrum, Jacob L. Edmondson, Christopher P. Wardell, Brittany G. Griffin, Sara C. Shalin, Murat Gokden, Issam Makhoul, Alan J. Tackett, Analiz Rodriguez

**Affiliations:** 1 Department of Biochemistry and Molecular Biology, University of Arkansas for Medical Sciences, Little Rock, AR 72205 USA; 2 Department of Biomedical Informatics, University of Arkansas for Medical Sciences, Little Rock, AR 72205 USA; 3 Department of Neurosurgery, University of Arkansas for Medical Sciences, Little Rock, AR 72205 USA; 4 Department of Pathology, University of Arkansas for Medical Sciences, Little Rock, AR 72205 USA; 5 Department of Medical Oncology, University of Arkansas for Medical Sciences, Little Rock, AR 72205 USA

**Keywords:** Melanoma, Brain metastases, Proteomics, Proteogenomics, Multi-omics, PIK3CG

## Abstract

Melanoma brain metastases (MBM) portend a grim prognosis and can occur in up to 40% of melanoma patients. Genomic characterization of brain metastases has been previously carried out to identify potential mutational drivers. However, to date a comprehensive multi-omics approach has yet to be used to analyze brain metastases. In this case report, we present an unbiased proteogenomics analyses of a patient’s primary skin cancer and three brain metastases from distinct anatomic locations. We performed molecular profiling comprised of a targeted DNA panel and full transcriptome as well as proteomics using mass spectrometry. Phylogeny demonstrated that all MBMs shared a SMARCA4 mutation and deletion of 12q. Proteogenomics identified multiple pathways upregulated in the MBMs compared to the primary tumor. The protein, PIK3CG, was present in many of these pathways and had increased gene expression in metastatic melanoma tissue from the cancer genome atlas data. Proteomics demonstrated PIK3CG levels were significantly increased in all 3 MBMs and this finding was further validated by immunohistochemistry. In summary, this case report highlights the potential role of proteogenomics in identifying pathways involved in metastatic tumor progression. Furthermore, our multi-omics approach can be considered to aid in precision oncology efforts and provide avenues for therapeutic innovation.

## Introduction

In various cancer patient cohorts, the development of brain metastases (BM) is present in 24–45% of patients. Melanoma is one of the most common primary cancers to lead to BM. It is expected that 100,350 melanoma cases will be diagnosed in the US in 2020 and 6850 will die with metastatic disease [1]. Of those, up to 40% develop melanoma brain metastases (MBM) and up to 80% of patients have evidence of MBM upon autopsy. MBM is the leading cause of death in these patients and portends a median survival of less than a year [2, 3]. More research is needed in understanding mechanisms of melanoma metastatic progression to the brain.

It is known that metastatic cancer cells continue to evolve following hematogenous spread and can acquire new mutations [4]. Genomic characterization of BMs and their respective primary tumors demonstrate potential clinically actionable genetic alterations are present in BM in approximately 50% of samples. However, even in a previously reported large cohort of 86 patients, only two anatomically distinct BM from the same patient were studied [5]. In another large cohort of BM, only two patients with multiple BM were profiled [6]. None of these patients had MBM, but molecular heterogeneity has been demonstrated in synchronous melanoma metastases to various organs [7], therefore analysis of these specimen types may reveal additional clinically actionable mutations for patients with advanced disease.

Transcriptomics and proteomics provide complementary information to standard genomic analyses allowing for a more representative view of the tumor phenotype and can therefore be used in precision oncology efforts [8]. Transcriptomics have identified immune and metabolic features of MBM as well as characterized the microenvironment [9, 10]. Using protein arrays, Chen et al. [11] analyzed 9 MBMs and 20 melanoma extracranial metastases to identify potential therapeutic targets. Proteomics has also been used in melanoma to identify diagnostic biomarkers, molecular pathways of pathogenesis and therapeutic response [12–15]. Protein level evidence of gene expression can delineate changes that are not always present at the genome level. Furthermore, proteins are often the targets of cancer based therapies making proteogenomics integral for the identification of therapeutic targets [16]. In this case report, we use a multi-omics approach (i.e. genomics, transcriptomics, and proteomics) to study three anatomically distinct MBM in a treatment naïve patient. We therefore utilized proteogenomics to identify potential pathways of metastatic progression in MBM development.

## Case presentation

**Fig. 1:**
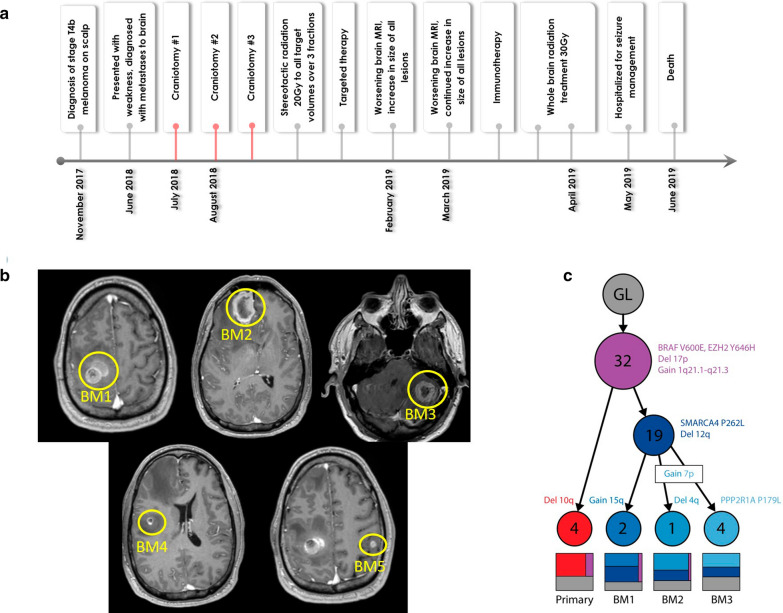
Clinical Course, Radiological Imaging and Phylogeny of Patient with 3 anatomically distinct melanoma brain metastases. **a** Timeline summarizing clinical course from diagnosis to death. Red lines denote the surgical resections during which tissue samples were obtained for proteogenomics. **b** Post contrast axial T1 MR images of Brain Metastases (BM) 1, 2, and 3, which were surgically removed. BM 4 and BM 5 were treated with radiation. **c** Tumor phylogeny shows the accumulated and shared mutations and copy number events gained during evolution from original germline (GL) cells to primary and metastatic tumors. Each node represents a new clonal lineage and is marked with the number of new SNVs observed. The treemaps show the proportion of each clone of which each sample was composed

A 46 year old Caucasian man with a previous diagnosis of stage T4b melanoma of the scalp 8 months prior, presented with a 2 week history of left hemiparesis and headaches (Fig. 1a). Magnetic resonance imaging (MRI) demonstrated 5 hemorrhagic metastatic brain lesions. Given his subacute hemiparesis and the presence of a metastatic lesion in the right motor strip, he had an awake craniotomy for removal of this lesion, which was confirmed to represent MBM by pathology. His hemiparesis improved and he did not receive brain radiation to the cavity or other intracranial lesions as planned. He was admitted 1 month later after his primary surgery with altered mental status and interval increase in his intracranial lesions. The right anterior frontal lesion now measured 3.6 cm (increased from 2.9 cm at presentation) with significant surrounding edema and associated localized brain compression. He had surgery for removal of this metastasis and again due to poor social support did not receive radiation. He came back within several weeks with headache, nausea, vomiting, and ataxia. Imaging again demonstrated progression of his intracranial lesions with compression of the fourth ventricle causing hydrocephalus by the left cerebellar lesion. He underwent a third craniotomy for resection of this symptomatic lesion and he was kept in the hospital to initiate brain radiation (Fig. 1b). Approximately 1 week after his last surgery, he began dabrafenib and trametinib. At his 2 month follow up, his MRI showed significant improvement in previously resected and previously radiated intracranial masses and no new intracranial masses. Six months after his last craniotomy, MRI demonstrated an increase in size of all lesions. The patient was also evaluated by oncology and it was determined he was not taking his chemotherapeutics as directed. Salvage whole brain radiation (30 Gy) was initiated the following month and he was started on immunotherapy (nivolumab and ipilimumab). Three months later the patient expired (Fig. 1a). Overall survival from his primary diagnosis was 18 months and survival from MBM diagnosis was 12 months.

Given the unique opportunity to study metachronous brain metastases in a patient who was treatment naïve at the time of surgeries, genomic, transcriptomic and proteomic profiling was performed as approved by our institutional review board. From the DNA sequencing data, the variant allele frequencies of all four tumors (i.e. the primary skin lesion and 3 BMs) were used to generate a phylogeny illustrating the evolutionary history of the tumors and the relative order in which single nucleotide variants (SNVs) and copy number variants (CNVs) occurred. Most SNVs and CNVs were shared by all samples, but a SMARCA4 mutation and deletion of 12q were shared by all BM and not the primary tumor (Fig. 1c).

We next used a proteogenomics approach to identify overlapping features amongst the DNA sequencing panel, transcriptome and proteome of all samples. 326 features overlapped between gene, transcript and protein in all 3 omic data sets and 8187 overlapping features were identified in both the proteomics and RNA sequencing data sets (Additional file 1: Fig. 1A). Substantial overlap was not seen in comparisons of gene transcripts to proteins between the primary tumor and each respective metastasis. Minimal overlap was observed between metastatic tumor comparisons (Additional file 1: Fig. 1B).

**Fig. 2:**
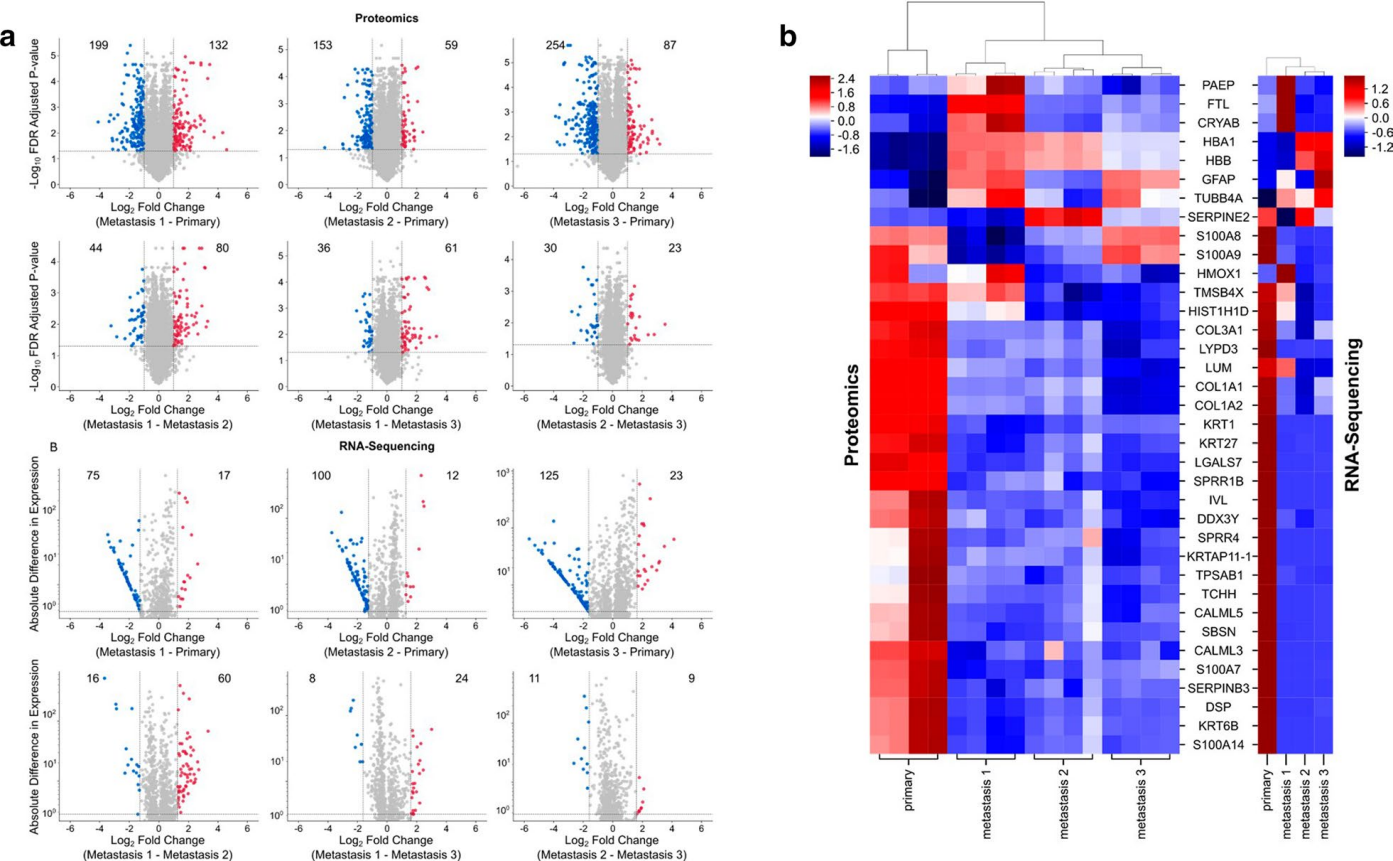
Proteogenomics demonstrate that MBMs cluster with each other. **a** Volcano plots of proteomics data. Proteins with fold changes significantly greater or less than 2 are highlighted as red and blue markers, respectively. **b** MD plots of RNA-sequencing data. Gene transcripts with probability > 0.90 are highlighted as red and blue markers, respectively. **c** Heatmap of differentially expressed proteins and genes that overlapped between the proteomics and RNA-sequencing data sets. Hierarchial clustering demonstrates that MBM cluster together and separately from the primary tumor. BM 2 and BM3 are the most similar amongst the MBM

To identify differentially expressed proteins amongst the tumors, we filtered protein expression by fold change (> 2) and *p* values (< 0.05). Using these cutoffs, significantly differential proteins that were down regulated or upregulated were represented in blue or red respectively. A large number of significantly different proteins were observed between the primary and each respective metastatic tumor but fewer differences in protein expression were present between the metastatic tumors, especially metastatic BM 2 versus 3 (Fig. 2a). RNA transcript profiles demonstrated similar trends as the protein expression (Fig. 2b).

**Fig. 3:**
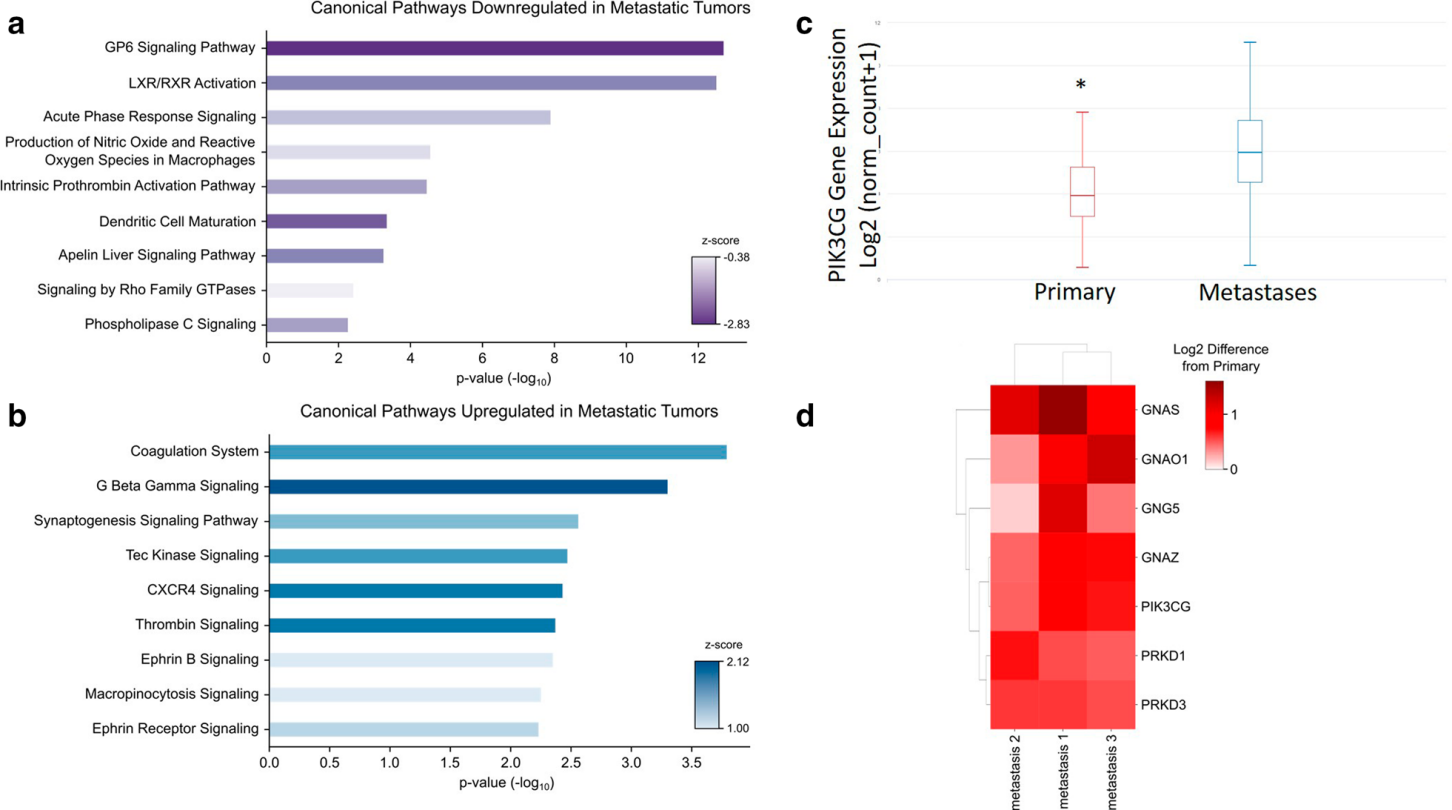
Pathway Analysis identifies PI3KCG as upregulated in MBM tissue. **a** Canonical pathways downregulated in the metastatic brain tumors. **b** Canonical pathways upregulated in the metastatic brain tumors. **c** PIK3CG expression in the cancer genome atlas melanoma cohort demonstrates significantly decreased expression in primary tumor tissue in comparison to metastatic tissue (*p* = 4.77 × 10^−15^). **d** Heatmap of proteins in common among the G beta gamma, CXCR4, and thrombin signaling pathways significantly increased levels of PI3KCG protein in the MBMs compared to the primary tumor

**Fig. 4:**
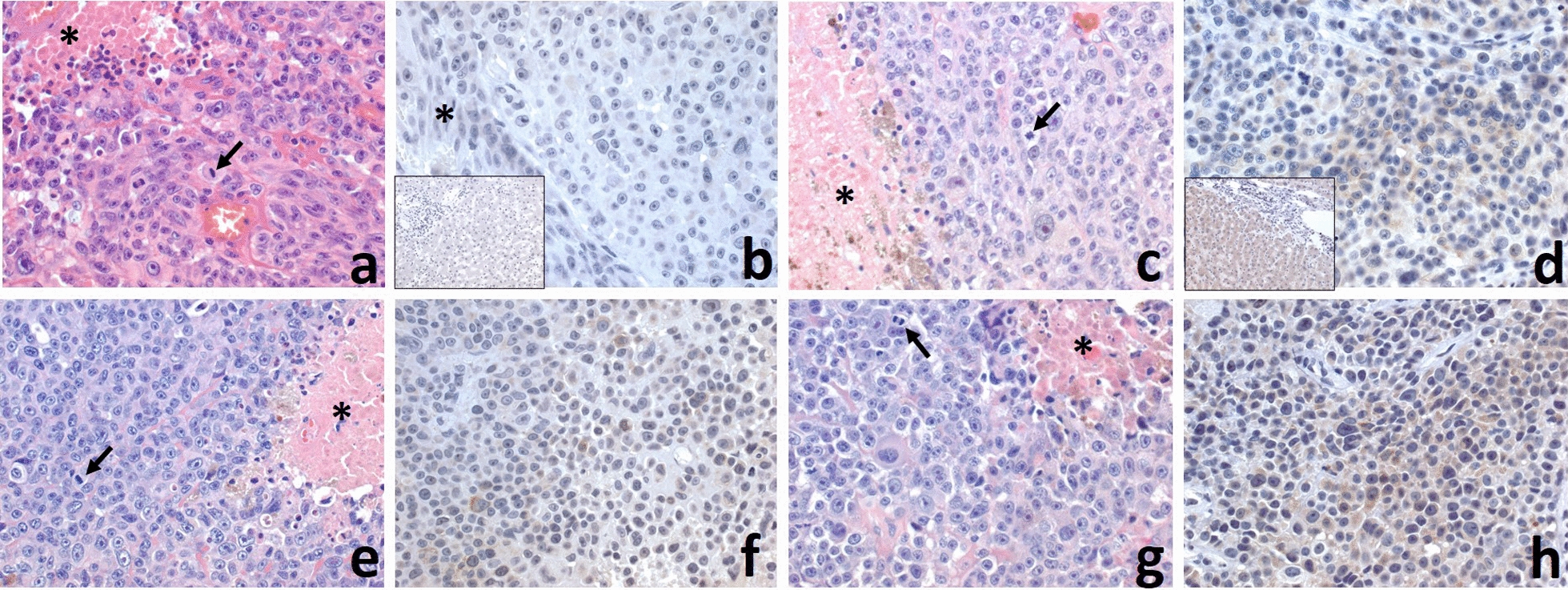
Comparison of histologic and PIK3CG immunohistochemical features of the primary skin and three metastatic lesions in the brain. All melanomas from the skin primary (**a**), 1st. (**c**), 2nd (**e**) and 3rd. (**g**) metastases in the brain were histologically similar with highly-atypical epithelioid malignant cells with prominent nucleoli, conspicuous cytoplasm, focal finely-granular green-yellow pigment, high mitotic activity (arrows), and necrosis (*). PIK3CG was negative in the skin primary (**b**; inset: negative control; *: epidermis), and focally and weakly positive in all three brain metastases (**d**; inset: positive control, **f** and **h**), with the 3rd metastatic lesion in the brain having a more widespread and stronger positivity (**h**), relative to others. (Original magnifications: **a**–**h**: 400 ×; insets: 200 ×)

Hierarchial clustering of significantly expressed gene transcripts and proteins demonstrated (protein expression: fold change ≥ 2 and *p* value < 0.05, RNA transcripts: probability > 0.90) that the primary tumor clustered separately from the metastatic tumors. Within the metastatic tumor cluster, BM 2 and 3 were the most similar (Fig. 2c). Ingenuity Pathway Analysis (IPA) was performed on the proteomics dataset to identify canonical pathways up and downregulated in the metastatic tumors. The nine most significant pathways downregulated in the metastatic tumors were predominately involved in immune response (Fig. 3a). On the other hand, the nine most significant canonical pathways increased in the metastatic tumor biopsies were most frequently associated with coagulation and G-protein signaling (Fig. 3b). The three significant pathways with the greatest z-score were G beta gamma signaling, CXCR4 signaling, and thrombin signaling (Fig. 3b). All three of these pathways were associated with GNAO1, GNAS, GNAZ, GNG5, PIK3CG, PRKD1, and PRKD3. Gene expression levels of top proteins identified in upregulated pathways from the metastatic samples were queried in the cancer genome atlas (TCGA). Phosphatidylinositol-4,5-bisphosphate 3-kinase catalytic subunit γ (PIK3CG) was identified in multiple upregulated pathways for the metastatic brain tumors and gene expression was significantly increased in metastatic melanoma samples in comparison to primary melanoma from TCGA data (Fig. 3c). PIK3CG is in the P2Y purigenic receptor signaling pathway and the protein level was upregulated in all three metastatic tumors as compared to the primary tumor (Fig. 3d). To validate this finding, we then performed immunohistochemistry (IHC) on histopathological tissue sections. All four tumors were histologically similar on hematoxylin and eosin staining (Fig. 4a, c, e, g). PIK3CG protein levels were significantly increased in metastatic tumors in comparison to the primary by proteomics. IHC confirmed no PIK3CG staining was present in the primary tumor and focally and weakly positive staining was present in all metastatic tumors (Fig. 4b, d, f, h).

## Conclusion and discussion

In summary, we used a multi-omics approach to characterize multiple MBM in a treatment naïve patient and were able to identify PIK3CG as a protein potentially correlated to metastatic progression. To our knowledge, our study is novel as an unbiased proteogenomic approach has yet to be applied to MBM samples matched to the primary tumor. Hierarchical analysis of transcriptomic and proteomic analyses confirmed that the individual MBM in anatomically distinct sites clustered similarly and had distinctive features separate from the primary tumor. We identified multiple pathways that were upregulated in the MBM tissue and identified PIK3CG as a protein present within many of these pathways. Furthermore, analysis of TCGA also confirmed PIK3CG gene expression to be significantly increased in their cohort of metastatic melanoma tissues (Fig. 3c). The PI3K (phosphoinositide 3-kinase) signaling pathway regulates numerous cell functions and has four isoforms of the catalytic subunits, p110α,-β,-δ,-γ, coded by PIK3CA,-B,-D and -G genes, respectively [17]. The PI3K pathway genes are important in melanogenesis with PIK3CG being a positive regulatory gene involved in the PI3K/Akt pathway [18]. Alteration of the PI3K/Akt pathway is a mechanism of BRAF inhibitor resistance and therefore is of clinical significance [19].

PIK3CG is a prognostic gene in melanoma with gene expression levels in primary and metastatic tissue correlating with survival [20]. Furthermore, PIK3CG and IL2RA genes were significantly associated with melanoma metastasis to the regional lymph node by analyzing TCGA data [21]. Interestingly, both of these genes are involved with inflammatory processes as PI3KCG is typically present on leukocytes like IL2RA [17]. Recent transcriptome analyses of melanoma patients with metastases demonstrated signatures consistent with immunosuppression in MBM compared to extracranial metastases [9]. However, this study did not include data from the primary tumor. In our case study, we did identify multiple differential inflammatory pathways in both the tumor and MBM tissue. Further characterization of the tumor immune microenvironment may be of interest in future studies with the incorporation of single cell sequencing.

PIK3CG was a protein of interest as it was present in multiple upregulated pathways in our MBM tissue. We choose to validate our findings with IHC and confirmed PIK3CG was increased in our MBM tissue in comparison to the primary tumor where it was absent. Previous proteomic array data of MBM and extracranial melanoma metastases implicated the PI3K pathway as a potential therapeutic target [11]. With our findings and the data of others from TCGA, PI3KCG may be implicated in metastatic tumor progression in melanoma. We plan to perform further testing on larger cohorts to determine the role of PI3KCG in brain metastases. Nonetheless, our proteogenomic platform is a useful approach to identify potential targets and to further the understanding of etiopathogenesis in MBM, a devastating disease process.

## Methods

Nucleic Acid Isolation. DNA and RNA sequencing were performed on the patient’s tumor specimens and saliva using the xT Laboratory Developed Test at Tempus’ Clinical Laboratory Improvement Amendments/College of American Pathologists-accredited laboratory in Chicago, IL. Tumor specimens were selected by the neuropathologist to ensure only tumor tissue was present. Tumor DNA was extracted from tumor tissue sections with tumor cellularity higher than 20% to exclude areas of necrosis and proteinase K digested. Total nucleic acid extraction is performed with a Chemagic360 instrument using a source-specific magnetic bead protocol. Total nucleic acid is utilized for DNA library construction, while RNA is further purified by DNaseI digestion and magnetic bead purification. The nucleic acid is quantified by a Quant-iT picogreen dsDNA reagent Kit or Quant-iT Ribogreen RNA Kit (Life Technologies), and quality is confirmed using a LabChip GX Touch HT Genomic DNA Reagent Kit or LabChip RNA High HT Pico Sensitivity Reagent Kit (PerkinElmer).

DNA Library Construction. One hundred nanograms of DNA for each tumor and normal sample was mechanically sheared to an average size of 200 base pairs using a Covaris ultrasonicator. The libraries were prepared using the KAPA Hyper Prep Kit. Briefly, DNA underwent enzymatic end-repair and A-tailing, followed by adapter ligation, bead-based size selection, and PCR. After library preparation, each sample was hybridized to a custom designed probe set. Recovery and washing of captured targets was performed using the SeqCap hybridization and wash kit. The captured DNA targets were amplified using the KAPA HiFi HotStart ReadyMix. The amplified target-captured libraries were sequenced on an Illumina HiSeq 4000 System utilizing patterned flow cell technology.

RNA Library construction. One hundred nanograms of RNA per tumor sample was fragmented with heat in the presence of magnesium to an average size of 200 base pairs. The RNA then underwent first strand cDNA synthesis using random primers, followed by combined second strand synthesis and A-tailing, adapter ligation, bead-based cleanup, and library amplification. After library preparation, samples were hybridized with the IDT xGEN Exome Research Panel. Target recovery was performed using Streptavidin-coated beads, followed by amplification using the KAPA HiFi Library Amplification Kit. The RNA libraries were sequenced to obtain approximately 65 million reads on an Illumina HiSeq 4000 System utilizing patterned flow cell technology.

DNA sequencing analysis. Sequencing reads were aligned to reference genome GRCh37 using Novoalign (there is no publication; just reference this link: http://www.novocraft.com/), followed by sorting and marking of duplicate reads. The germline sample was sequenced to 580 × depth and the tumors varied between 1000 × and 2000 × depth. Single nucleotide variants (SNVs)and indels were called using Strelka2 [22]. Further filtering was performed using the default settings ofFiNGS (https://github.com/cpwardell/FiNGS). All variants were manually inspected by an experienced bioinformatician using IGV [23]. The sites of any variants that passed filters in any sample were analyzed in every sample, to ensure that lower-quality or subclonal variants were not treated as false negatives. Filtered variants were annotated using Ensembl VEP [24]. Structural variants were called using Manta [25], copy number variants were called using CNVkit [26] and all putative variants were manually reviewed. The tumor phylogeny was reconstructed using LICHeE [27].

RNA-sequencing Data Analysis. The RNA reads were checked for quality of sequencing using FastQC v.0.11.8. The adaptors and low-quality bases (Q < 20) were trimmed to a minimum of 36 base pairs using Trimmomatic v0.39. Reads that passed quality control were aligned to the Homo sapiens GRCh38 reference genome using hisat2 [28]. The read alignments were then assembled into transcripts using StringTie (version 1.3.6) [29]. NOISeq was used for quality control and analysis due to the lack of replicates in the experimental design [30, 31]. The prepDE.py (http://ccb.jhu.edu/software/stringtie/dl/prepDE.py) python script was used to prepare the input matrix of read counts mapped to particular genomic features required for NOISeq directly from the files generated by StringTie. The ensemble and entrez IDs were obtained using the UseMart function from Biomart [32, 33]. We applied the NOISeq-sim function to simulate technical replicates using the following parameters: pnr = 0.2, nss = 5, and v = 0.02. Genes with low counts were removed prior to downstream analysis. The filtered dataset was normalized for compositional bias using trimmed mean of M values (TMM) and batch corrected to account for different sequencing days. Pair-wise comparisons were analyzed between the primary tumor and each of the three metastatic lesions as well as between each of the metastatic lesions independently of the primary tumor. Genes were considered to be significant with a probability > 0.90.

## Immunohistochemistry

FFPE tissue blocks were cut into 5-μm sections, which were stained with PIK3CG (Invitrogen, Carlsbad, CA; catalogue number MA5-26087) according to the manufacturer’s instructions. Liver sections were used as a positive control. Stained slides were reviewed by a neuropathologist (M.G.) to determine the average intensity of staining. Slides with insufficient viable tumor tissues were excluded from analyses.

TCGA Analysis. The University of California Santa Cruz Xena platform was used to interrogate the TCGA database. We queried the melanoma cohort to identify the gene expression levels for PIK3CG in primary and metastatic melanoma tissues [34].

Tissue Processing for proteomics. Two 10 µm scrolls were cut from FFPE patient tissue blocks and deparaffinized according to the protocol used by Hughes et al. [35]. 1 mL xylene was added to each sample and vortexed for 10 s. Samples were centrifuged for 3 min at 15,000 g and the supernatant removed. The tissue was then resuspended in 1 mL 100% ethanol, vortexed for 10 s, and centrifuged at 15,000 g for 3 min. The supernatant was discarded, and the samples were air dried for 10 min. The deparaffinized samples were resuspended in 100µL lysis buffer (100 mM Tris, 2% SDS, pH 7.6) and heated for 30 min at 95 °C followed by water bath sonication for 5 min on high with a Diagenode Bioruptor. After sonication sample were incubated overnight at 65 °C. Following the overnight incubation, a BCA protein assay kit (Pierce) was used to determine protein concentration for each of the samples.

Filter Aided Sample Preparation (FASP) and Tandem Mass Tag (TMT) labeling. 100 µg of each tumor sample was reduced using TCEP at a final concentration of 10 mM for 30 min at 37 °C. The samples were diluted in 11 times the volume of UA buffer (8 M urea, 100 mM Tris, pH 8.5). The diluted samples were passed through FASP filters (30,000 MWCO, Sartorius Vivacon 500 DNA Concentrator VN01H22). Samples were washed with UA buffer and alkylated with iodoacetamide. Following treatment with iodoacetamide, the samples were washed with UA buffer and 50 mM TEAB. Samples were then trypsinized into peptides at 37 °C overnight. The samples were desalted (SEP-PAK C18 Cartridge, Waters), lyophilized, and stored at − 20 °C. The peptides were TMT labeled (ThermoFisher) according to the manufacture’s protocol.

Fractionation and Mass Spectrometry. The TMT-labeled samples were processed in two batches containing all 4 samples in technical duplicate. The first batch was fractionated off-line with acidic pH followed by basic pH reverse-phase chromatography on a 100 × 1.0 C18 column (Acquity BEH, Waters) and combined into 48 super fractions. The second batch was fractionated off-line with only basic pH reverse-phase chromatography on a 100 × 1.0 C18 column (Acquity BEH, Waters) and combined into 34 super fractions. The super fractions from each batch were then separated by reverse phase resin (Jupiter Proteo resin, Phenomenex) and resolved in-line on a 200 × 0.075 mm column using a UPLC system (nanoAcquity, Waters) coupled with a Thermo Orbitrap Fusion Tribrid mass spectrometer. Peptides from both batches were eluted with a 60-min gradient from 97:3 to 67:33 buffer A:B ratio (buffer A: 0.1% formic acid, 0.5% acetonitrile; buffer B: 0.1% formic acid, 99.9% acetonitrile). The peptides were ionized by electrospray (2.15 kV) followed by mass spectrometric analysis (Orbitrap Fusion Tribrid mass spectrometer, Thermo) using multi-notch MS3 parameters.

Mass Spectrometry Data Analysis. Data obtained from the mass spectrometer was analyzed using MaxQuant (Max Planck Institute) to identify proteins and quantify reporter ions with a parent ion tolerance of 3 ppm, a fragment ion tolerance of 0.5 Da, and a reporter ion tolerance of 0.001 Da. MS3 reporter ion intensities of each batch were normalized to the median of the samples. The reporter ion intensities were then normalized by individual proteins to the protein median across batches. Significance was calculated in GraphPad using an unpaired t-test with Benjamini, Krieger, and Yekutieli multiple t-test correction with an FDR (Q) = 5%.

## Supplementary information

**Additional file 1: Figure S1.** Comparison of different omics data sets. **A** A Venn diagram of features identified that overlap between proteomics, RNA sequencing, and DNA sequencing. **B** Venn diagrams of significantly different features in proteomics and RNA sequencing.

## Data Availability

The datasets generated or analysed during the current study are not publicly available due to protection of individual privacy/genetic data protection, but are available after anonymization from the corresponding author on reasonable request.
