# Supplementary figures and images for: Proteogenomic analysis of melanoma brain metastases from distinct anatomical sites identifies pathways of metastatic progression
Source: Acta Neuropathol Commun. 2020 Sep 5;8:157. doi: 10.1186/s40478-020-01029-x (PMC7487560; doi:10.1186/s40478-020-01029-x)

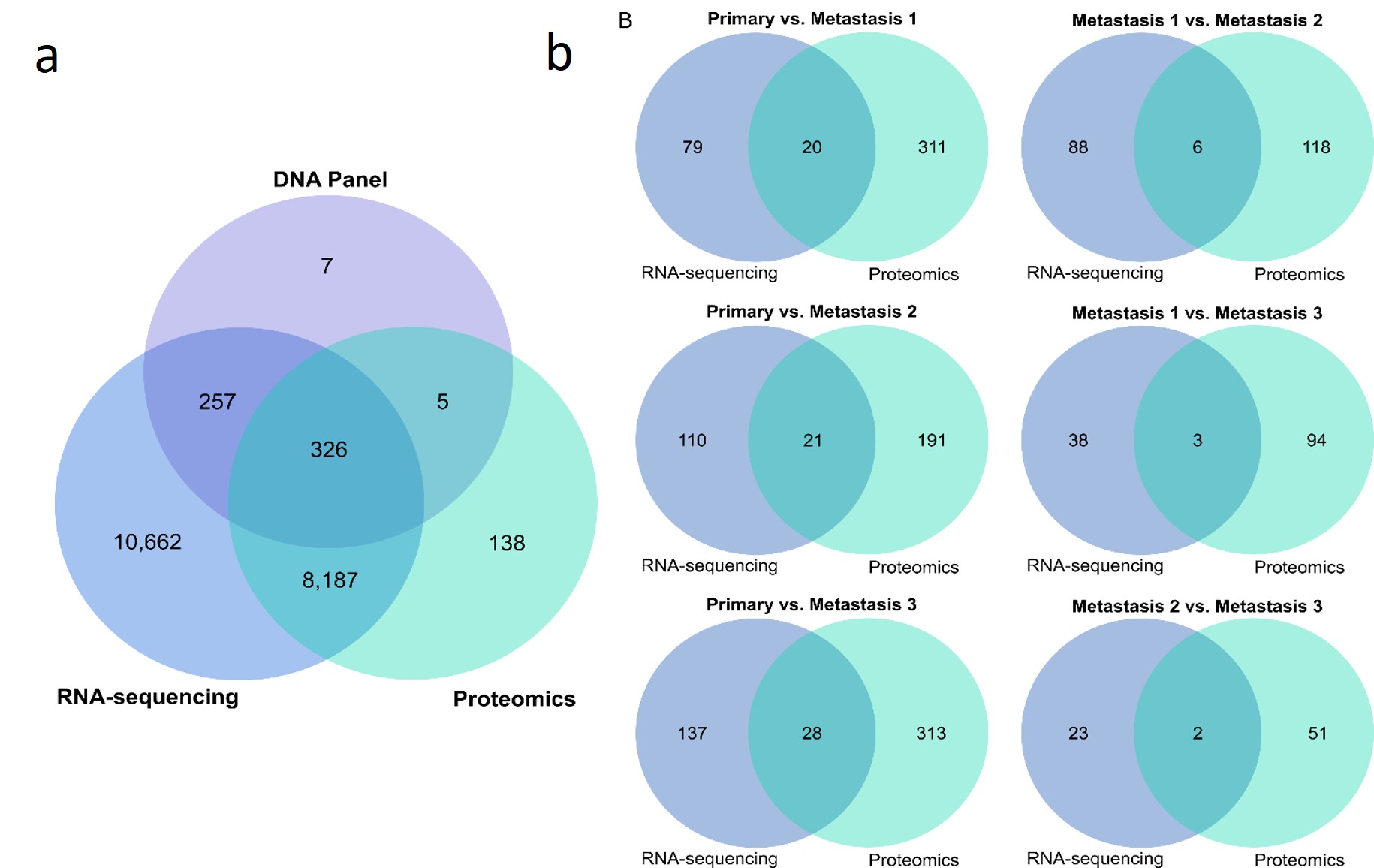

Supplement: Supplementary file 1 — Additional file 1: Figure S1. Comparison of different omics data sets. A A Venn diagram of features identified that overlap between proteomics, RNA sequencing, and DNA sequencing. B Venn diagrams of significantly different features in proteomics and RNA sequencing. [file 40478_2020_1029_MOESM1_ESM.jpg]
